# Supplementary material for: Correlative single‐molecule localization microscopy and electron tomography reveals endosome nanoscale domains
Source: Traffic. 2019 Jul 11;20(8):601–17. doi: 10.1111/tra.12671 (PMC6771687; doi:10.1111/tra.12671)
Supplement: Supplementary file 1 — Appendix S1 Supporting Information [file TRA-20-601-s001.pdf]

Correlative SMLM and electron tomography reveals endosome nanoscale domains

Christian Franke [1*], Urska Repnik [1*], Sandra Segeletz [1*], Nicolas Brouilly [1,2*], Yannis Kalaidzidis [1,3], Jean-Marc Verbavatz [1,4] and Marino Zerial [1]

[1]Max-Planck-Institute of Molecular Cell Biology and Genetics, Pfotenhauerstr. 108, 01307 Dresden, Germany.
[2]Instutut de Biologie du Developpement de Marseille-Luminy, Aix-Marseille Universite, Marseille, France
[3]Faculty of Bioengineering and Bioinformatics, Moscow State University, Moscow, Russia
[4]Institut Jacques Monod, CNRS, Université Paris-Diderot, Sorbonne Paris Cité, 75013 Paris, France.
* authors contributed equally
Corresponding author: Marino Zerial;

Max Planck Institute of Molecular Cell Biology and Genetics, MPI-CBG, Pfotenhauerstrasse 108, 01307 Dresden, Germany; Tel.: +49-351-210-1100 ; Fax: +49-351-210-1389 ; zerial@mpi-cbg.de

Supplementary Note

Our superCLEM workflow largely relies on a multi-step sample preparation, mainly to allow performing the heavy metal staining required for EM after the successful SMLM imaging. Along these steps we recognized a number of possible pitfalls leading to the destruction of the sample. Furthermore, due to the unique set of requirements demanded from the sample for superCLEM we think it to be important to elude on some finer details of the analysis.

Compared to the optimal case of Tokuyasu sections directly deposited on glass, sections applied to EM grids proved to be more challenging for SMLM imaging. This was because the formvar film supporting the sections cannot be completely flat, hence

only a small region at a time was in focus. Additionally, because this setting often produced a slight gap between the section and the glass, we could not always apply a full TIRF illumination, leading to reduced overall SMLM quality. Furthermore, the copper EM-grid and the active contents of the photo-switching buffer, probably mainly the thiols, led to a progressing change in pH-value and thiol concentration during acquisitions, leading to suboptimal conditions for SMLM after the first measurement. We tried to circumvent this issue by using gold EM-grids but observed similar effects, probably due to progressing adhesion of thiols to the extended gold surface of the grids. Consequently, the SMLM quality, regarding photo-switching and single-molecule brightness, in superCLEM is slightly inferior compared to sections directly deposited on glass. For future endeavours the use of intrinsically blinking high-performance dyes could further improve the method.

Drying the back-side of a grid with the filter paper after SMLM imaging is probably the most delicate step of the whole protocol with great impact on the ultrastructural preservation. If the front side of a section is accidentally exposed to drying (in the absence of methylcellulose), its ultrastructure will be irreversibly destroyed. Additionally, the formvar film can get torn during this procedure. Especially during the early stages of the protocol development we lost a significant portion of samples around this step. We assure potential users that with some patience and practice this step can be performed with sufficient success rates, although a certain loss ratio will remain.

Due to intrinsic restrictions of the individual methods may cause that signals cannot fully or not certainly be assigned to an underlying ultrastructure. These restrictions include the finite angular range (‘missing wedge’) in electron tomography, shrinkage of sections after drying and the correlation of 2D fluorescence data with 3D electron tomography.
We experience the missing wedge as a problem especially for tubular structures, as it hard to observe their connections with the central vesicles due to the limited information that can be acquired by tomography.

On the other side, we could also observe tubular structures associated with the central vesicle of an endosome that did not have an obvious counterpart in the SMLM image (e.g. Supplementary Figure 7). Given the density of SMLM Tfn signal in tubules, these most likely represent other recycling tubules that contain different cargos or tubules that are destined for retrograde transport.

We observed a significant axial shrinkage of sections after drying, estimating a remaining 1/3 of their initial thickness. This allowed us to analyse sections that were originally thicker than the commonly used 300 nm, though it is likely that some distortions to the ultrastructure took place. Tokuyasu sections are so far not frequently used for electron tomography and the structural collapse along a z-axis is probably the main reasons. On the positive side, the membranes are well preserved and appear clearly white due to the negative staining. As pointed out in the main manuscript, the delicate structures of tubules originating from the globular part of endosomes are consistent with resin embedding, where no drying and the connected axial shrinkage can take place. So we are confident that shrinkage will lead to ultrastructural compression rather than major distortion, but users have to keep in mind that this may not be the case for alternative target structures.

Finally, the correlation of two-dimensional SMLM data to the three-dimensional EM tomograms is only possible under certain conditions. The usage of densely seeded fiducial markers in the imaging plane allows the mapping of SMLM data to the ultrastructure with nanometer precision laterally, but not axially, where signals can be assigned to certain likely structures. As illustrated in Suppl. Figure S5, the lateral overlay precision based on the fiducials seems limited to approximately 40 nm. This is true for the specific setting of this quantification, i.e. aligning a large field of view as illustrated in Suppl. Figure 8. We called this step the rough alignment in the main manuscript which is mainly used to pinpoint the endosome of interest with certainty. Therefore, the before stated 40 nm can be seen as an absolute lower bound for the overlay accuracy regarding the final alignment, which consists of a composite of the initial, fiducial based overlay and a subsequent manual refinement. Distinct structures

like ILVs and singular tubules can be interpreted as intrinsic fiducial structures and allowed us to fine-tune the lateral and angular positioning of the SMLM signal, without changing the scale of the SMLM signal.

Concordantly, based on *a priori* knowledge about the proclivity of Tfn to reside in recycling tubules and EGF in ILVs we experience that after endosome segmentation particular cargo could be assigned to particular compartment with certain probability. Adapting the workflow to three-dimensional SMLM will allow a complete correlation between imaging modi, but potential users should be aware of chromatic shifts and other artefacts that complicate multi-colour three-dimensional SMLM. Alternatively, one could use ultra-thin Tokuyasu sections to virtually eliminate the possibility of axial uncertainties. In the case of tubulo-vesicular morphology of endosomes, we found that 100 nm thick sections do not contain sufficient ultrastructural information to reliably reconstruct high-quality SMLM images of endosomes (especially tubules). In our estimate, the usage of thin sections has potential for compact organelles or more uniform distribution of the molecules of interest.

Supplementary Information

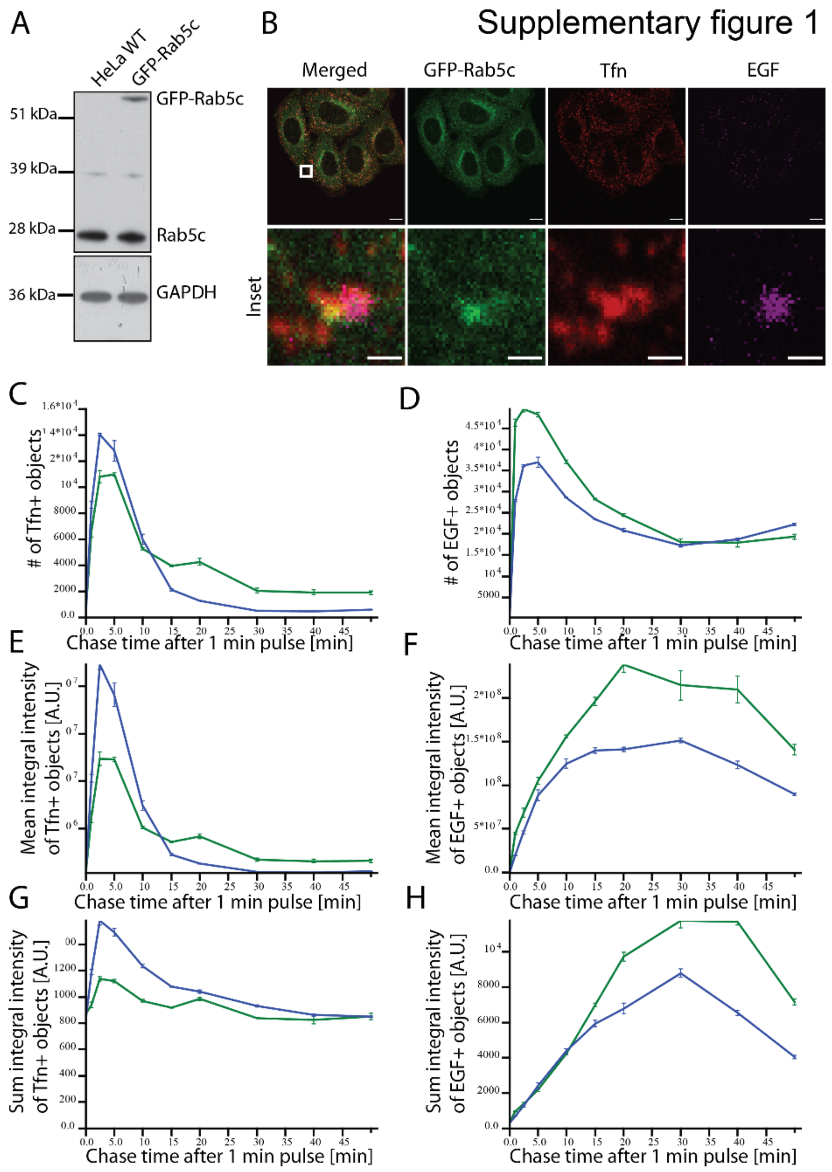

Figure S1: Validation of the GFP-Rab5c BAC HeLa cells.
(A) Immunoblot of GFP-Rab5c cells with high GFP-Rab5c expression with an anti-Rab5c antibody. The GFP-tagged protein has an expected size of ~50 kDa. The percentage of each population was 30% relative to the total Rab5c amount. The GAPDH band was used as loading control.
(B) Immunofluorescence of GFP-Rab5c cells. Cells were fed with Tfn and EGF continuously for 15 min before fixation. Displayed is a representative image of the cells (upper panel) with an Inlay (lower panel) that is the enlargement of the white rectangle. Confocal images were analysed using the Fiji software. Scale bar: 10 µm, Inlay: 1 µm

(C-H) Kinetics of Tfn (C, E and G) and EGF (D, F and H) trafficking by the GFP-Rab5c BAC cell line (green) and control HeLa (blue) (Mean ± SEM)
(C-D) Number of Tfn- (C) and EGF-positive (D) endosomes per masked area.
(E-F) Mean integral intensity of Tfn- (E) and EGF-positive (F) endosomes per masked area.
(G-H) Sum integral (overall) intensity of Tfn- (G) and EGF-positive endosomes (H) per masked area.

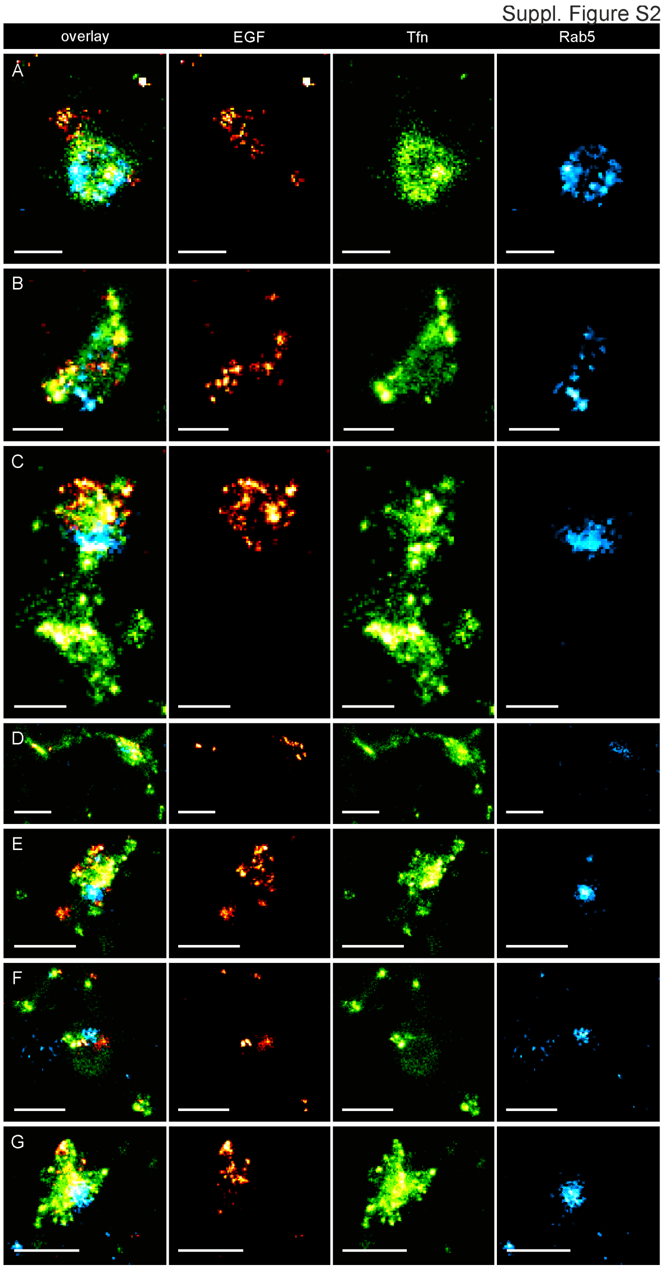

Figure S2: Additional examples for triple-colour whole cell SMLM

Triple-Colour Single-Molecule Localization Microscopy of endosomes in HeLa cells reveals the compartmentalisation of early endosomes. A-D Representative examples of endosomal structures displaying various types of compartmentalization of EGF (AF647, red), Transferrin (AF568, green) and Rab5c (Dronpa, cyan). Scalebar: 250 nm (A-C), 500 nm (D-G).

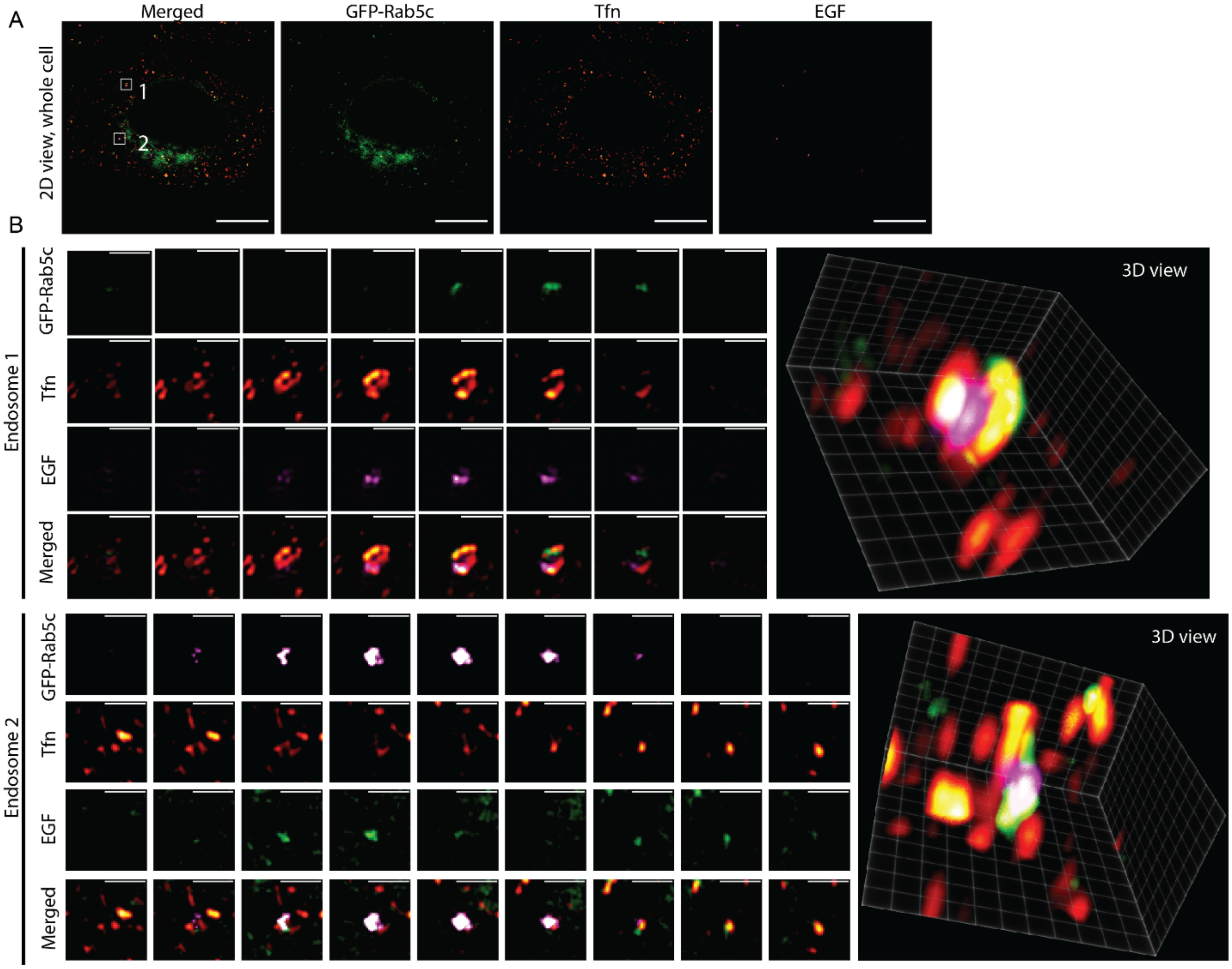

Figure S3: 3D-Structured illumination microscopy supports compartmentalization of Rab5c and cargo on endosomes.

3D-SIM characterization of Rab5c early endosomes positive for Tfn and EGF. GFP-Rab5c-labelled were fed with Alexa-568 Tfn and Alexa-647 EGF for 15 mins, fixed and then analysed with SIM. A. 2D view of whole cell expressing GFP-Rab5c (Scale bar: 10µm). B. Individual endosomes (enlargements from rectangle 2D view) marked by a white rectangle were enlarged and individual channels are shown in the direction of the plasma membrane facing the coverslip upwards (voxel size: 0.04, 0.04, 0.125 µm). Cytosolic background staining from GFP-Rab5c are a consequence of inactive Rab5c residing in the dense perinuclear area. Volume rendering (3D view) from the individual endosome images were generated using the ClearVolume software[1], Size of displayed grid: Endosome 1: 1µm x2.08 µm x2.08 µm; Endosome 2: 1.25 µm x2 µm x2 µm. Scalebar: 10 µm and 1 µm in the enlargements.

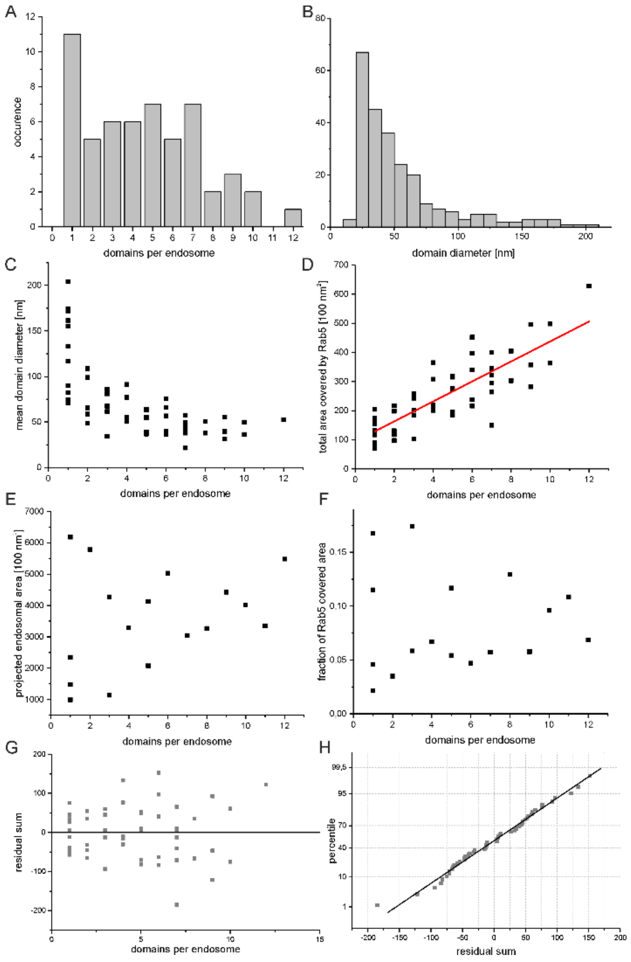

Figure S4. Morphological analysis of Rab5c domains in triple positive endosomes.

(A) Number of Rab5c domains per triple-positive endosome (4.9+-3.1, mean+-std).
(B) Distribution of Rab5c domain diameter. (55.1+-37.8nm, mean+-std).
(C) Mean Rab5c domain diameter dependent on the number of Rab5c domains within the endosomeMean Rab5c domain diameter dependent on the number of Rab5c domains within the endosome.
(D) Total endosomal area covered by Rab5c domains dependent on the number of Rab5c domains within the endosome. The linear fit has a R value of 0.82895 (extracted from OriginPro2017) and its residuals are displayed in (G) and (H).
(E) Dependency of the number of endosomal Rab5c domains and the projected (from cargo SMLM signal) total endosomal area. Endosomes, exhibiting a larger number of Rab5c domains tend to be of larger overall size.
(F) The fraction of projected endosomal area covered by Rab5c is virtually independent of the number of Rab5c domains.
(G) & (H) The residuals of the linear fit in (D) do not show any systematic divergence, thus indicating the validity of a linear model.

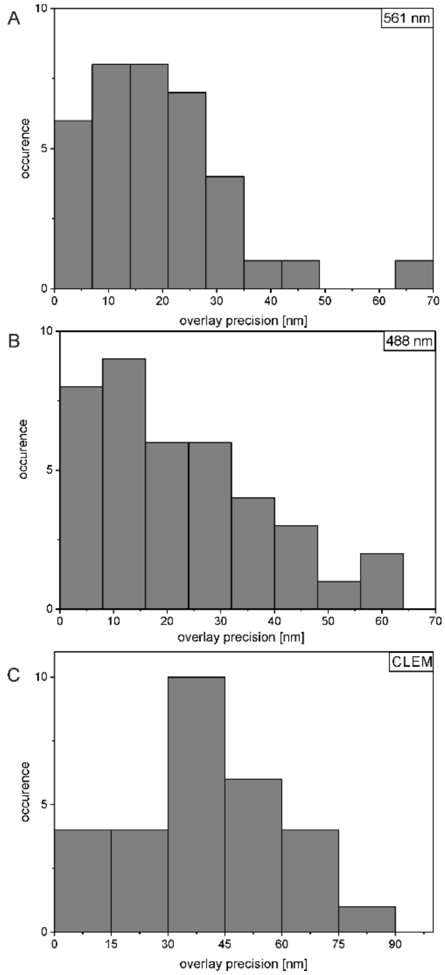

Figure S5: Multi-colour and SMLM to EM overlay precision based on bacterial fiducial markers. Distribution of distances measured after the alignment of the 561 nm SMLM channel (A) or the 488 nm SMLM channel (B) to the 647 nm SMLM reference channel. On average, an overlay precision of 19.6 ± 13.0 nm (mean±std) (17.8 ± 9.2 nm (median±mad)) and 22.7 ± 16.0 nm (mean±std) (19.8 ± 10.2 nm (median±mad)) for the 561 nm and 488 nm channel was determined respectively. The average SMLM to EM overlay precision (C) was determined to 39.4 ± 19.3 nm (mean±std) (38.7 ± 15.3 nm (median±mad)). In all cases, 5 independent field of views were analysed with N = 36; 39, 28 independent fiducial markers.

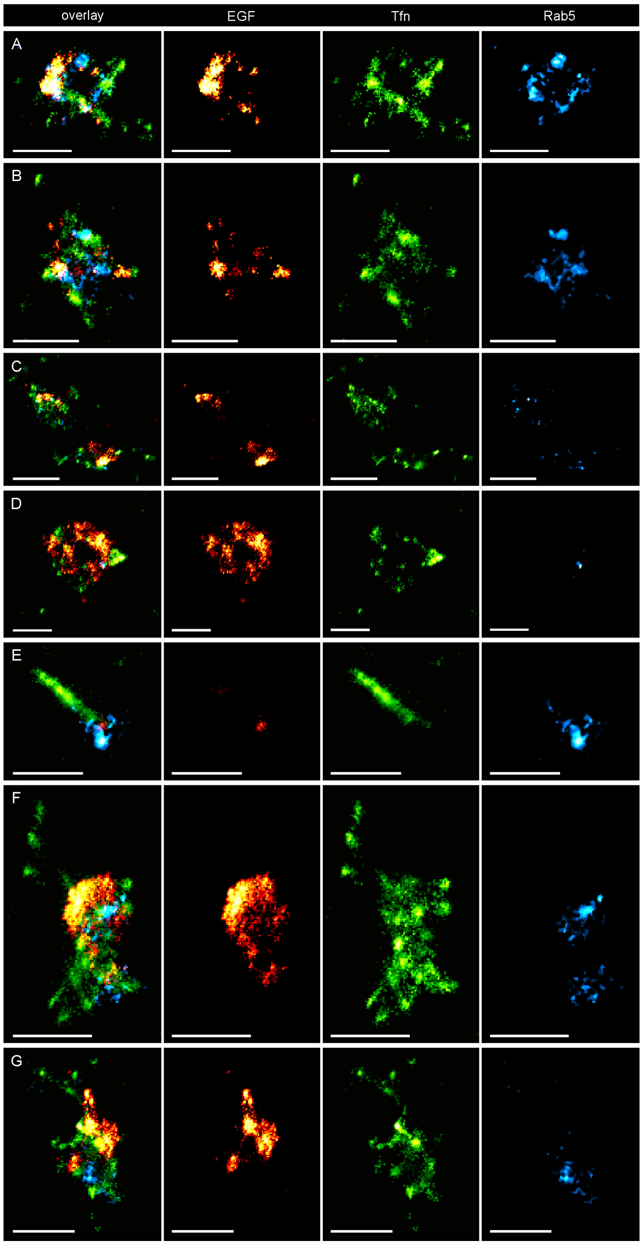

Figure S6: Additional examples for triple-colour SMLM on Tokuyasu sections

Triple-Colour SMLM of endosomes on Tokuyasu sections shows compartmentalization of early endosomes consistent with the analysis on whole cells. A-D Representative examples of endosomal structures displaying various types of compartmentalization of EGF (AF647, red), Transferrin (AF568, green) and Rab5c (Dronpa, cyan). Scalebar: 500 nm.

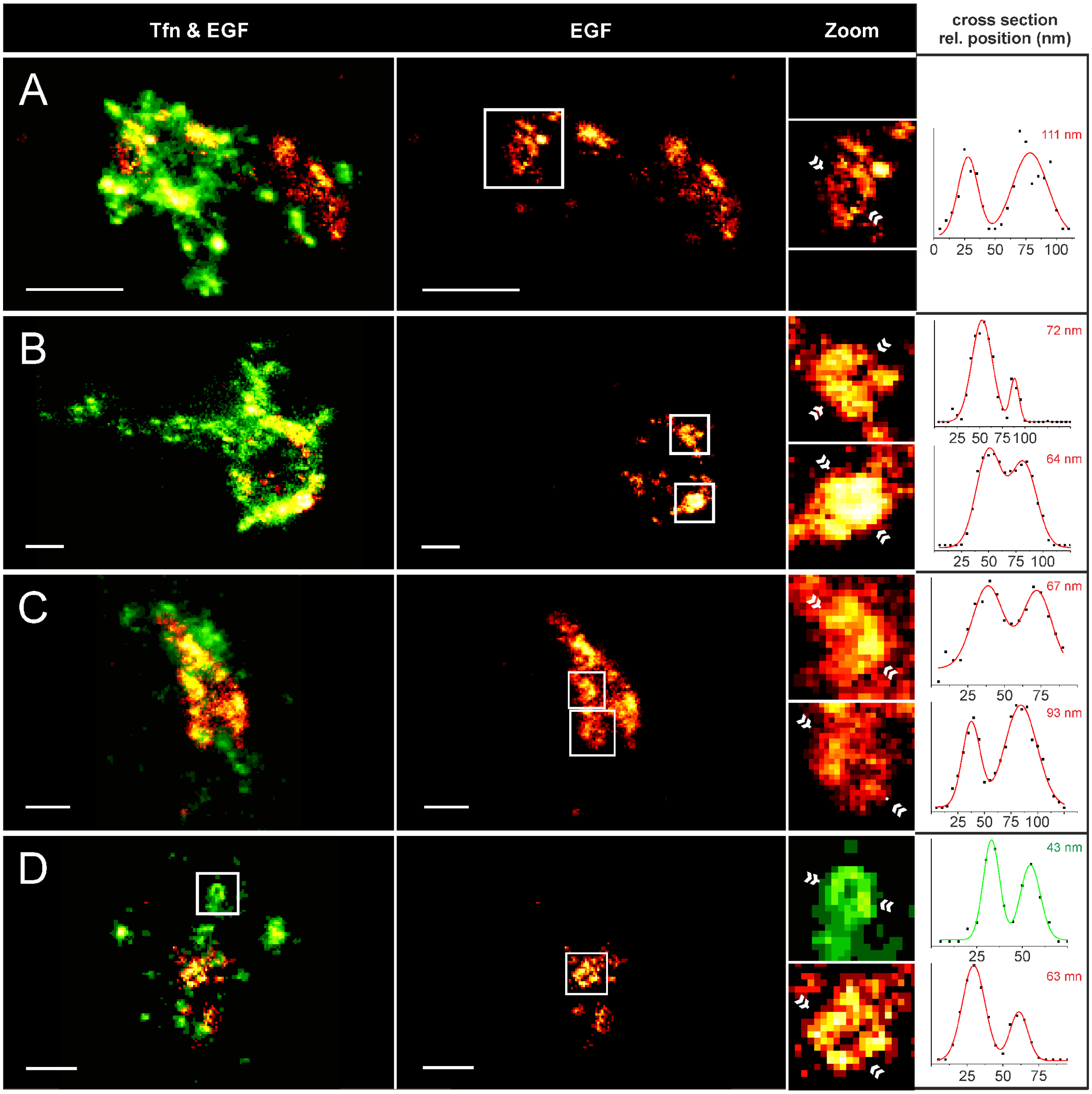

Figure S7: Distinct small EGF ring structures can be observed by SMLM on Tokuyasu sections. Original areas from zoom images are indicated as white boxes, while line-profile traces are indicated as white arrows. Black squares in line-profiles indicate data points, red, solid lines bimodal fits to the data. The peak-to-peak distances are indicated as coloured values in every line-profile. Scalebar: 250nm.

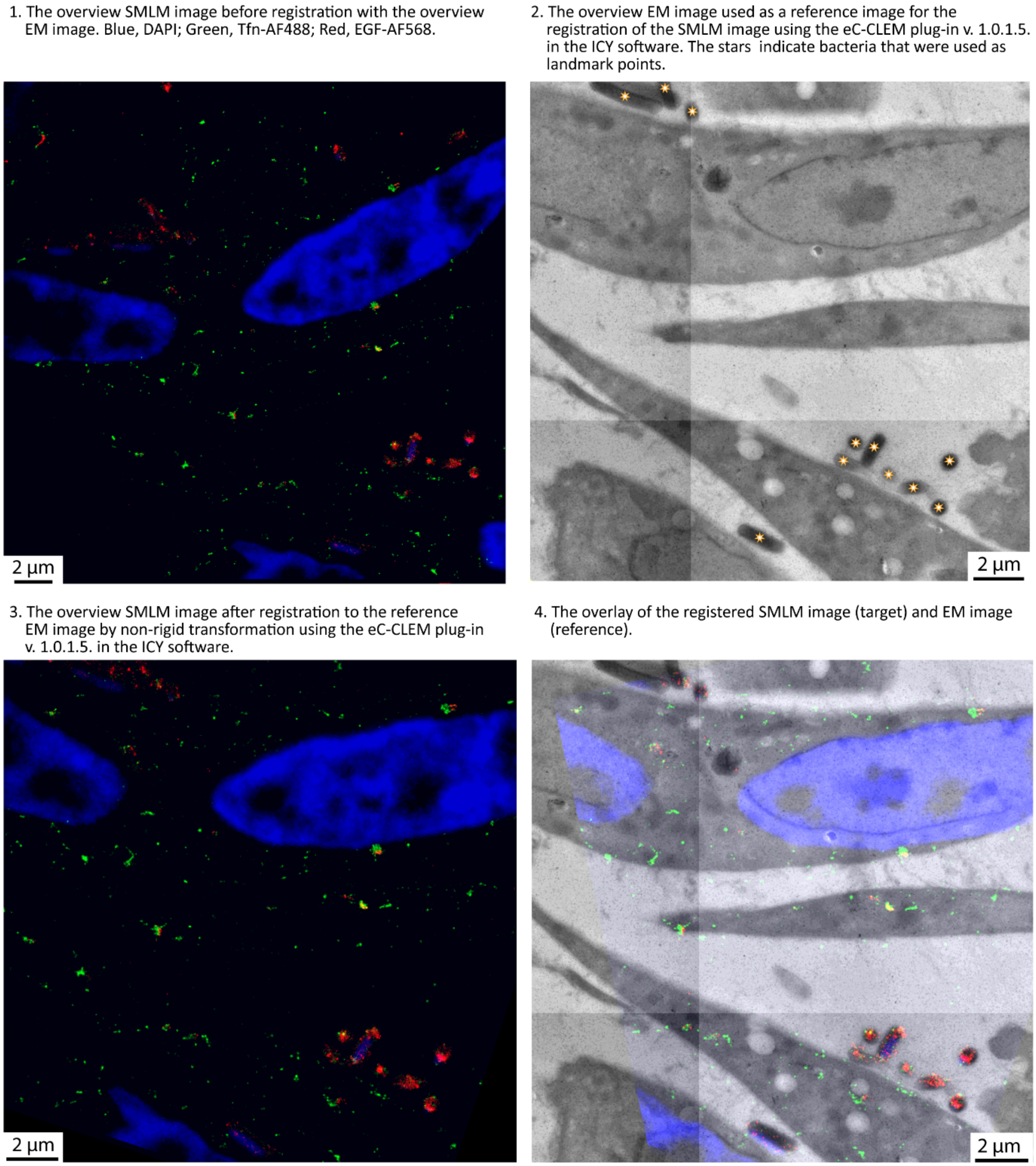

Figure S8: Registration procedure (overview).
Illustration of the registration procedure of the SMLM and the EM overview images using fluorescent bacteria (indicated with stars on the upper right image) as fiducials and the ICY software.

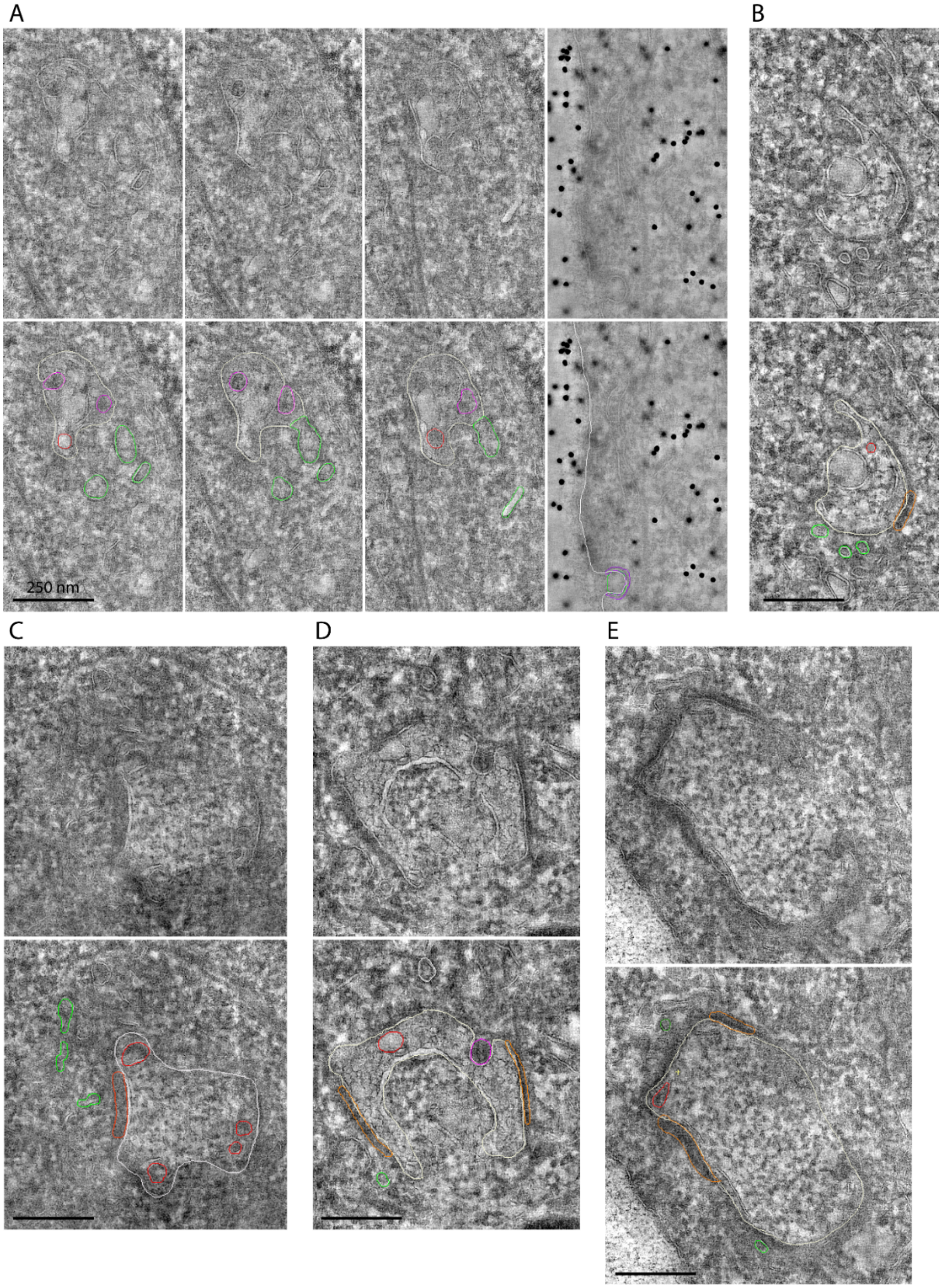

Figure S9: Examples of endosome segmentation in five different tomograms (A-E). Colour lines indicate structures that were segmented: (grey) endosome limiting membrane, (green) tubular structures, (red) intraluminal vesicles - ILVs, (magenta) ILV continuous with the limiting membrane, (orange) bilayered clathrin coats representing sorting microdomain on the limiting membrane, (purple) clathrin-coated vesicle.

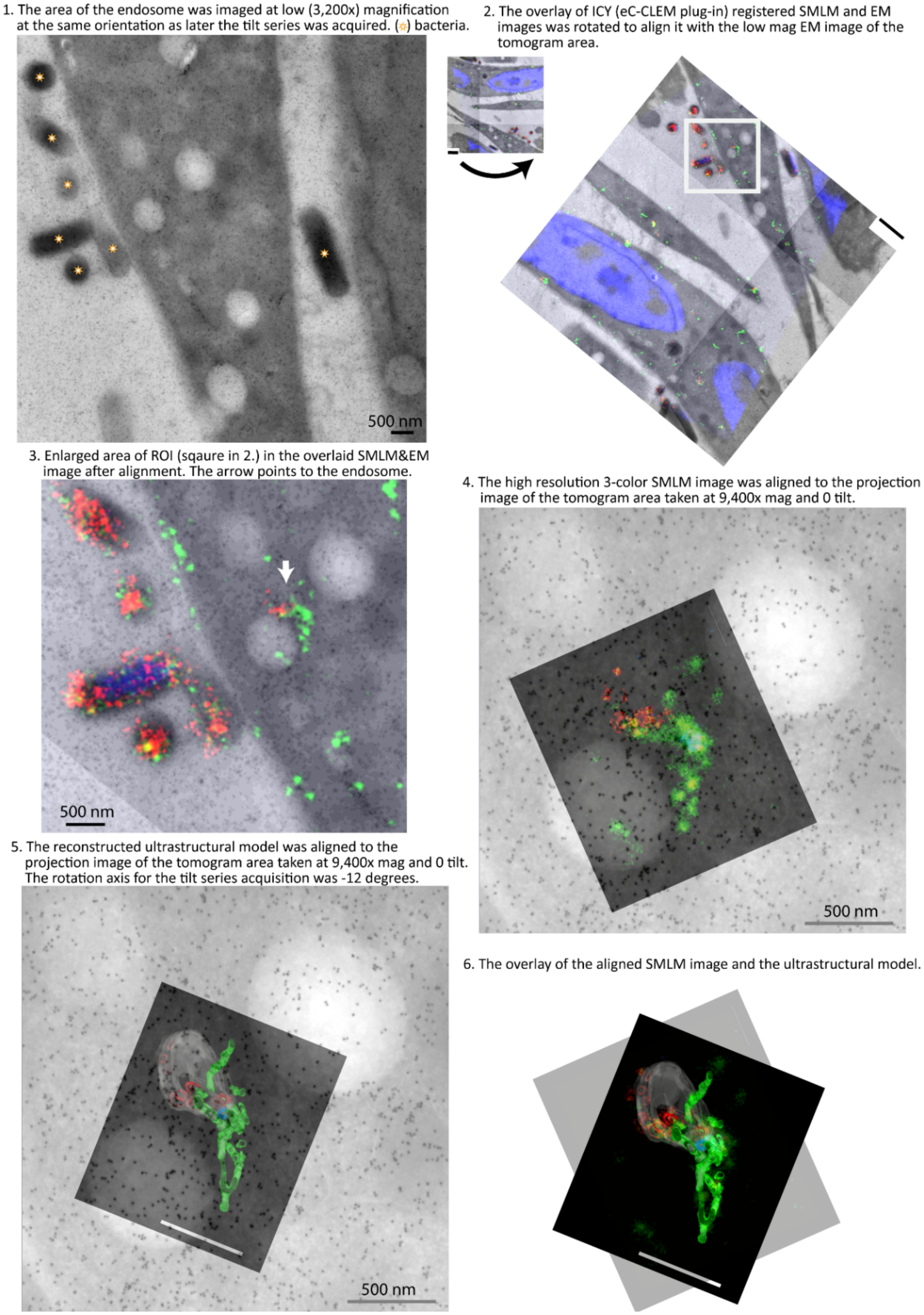

Figure S10: Registration procedure (endosome).
Illustration of the alignment procedure of the SMLM image and the ultrastructural model.

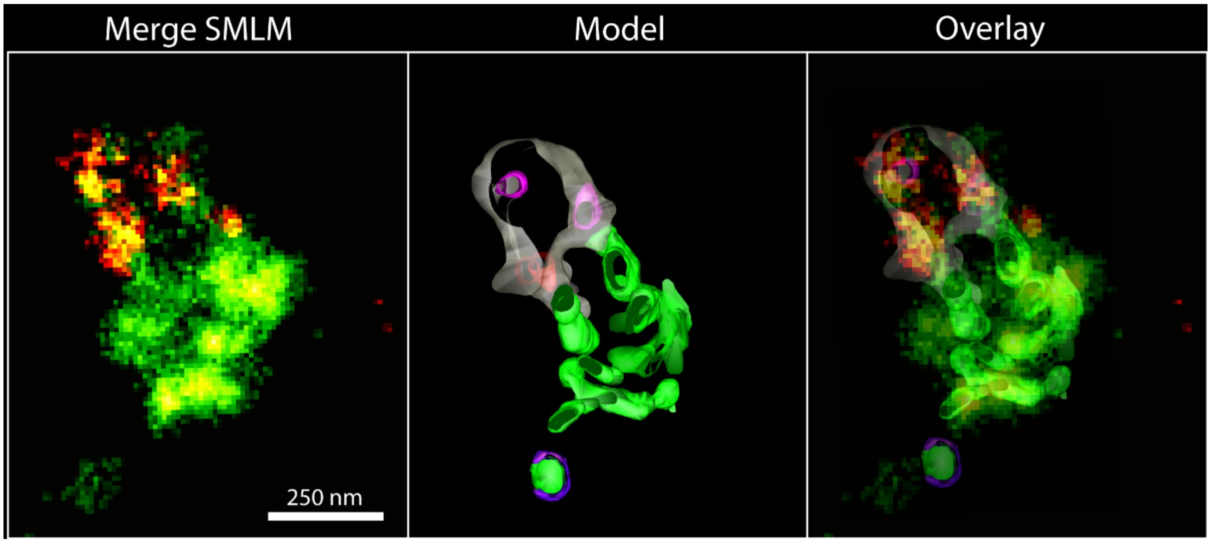

Figure S11: Direct overlay of the merged SMLM image and the coloured ultrastructural model presented in Figure 5.

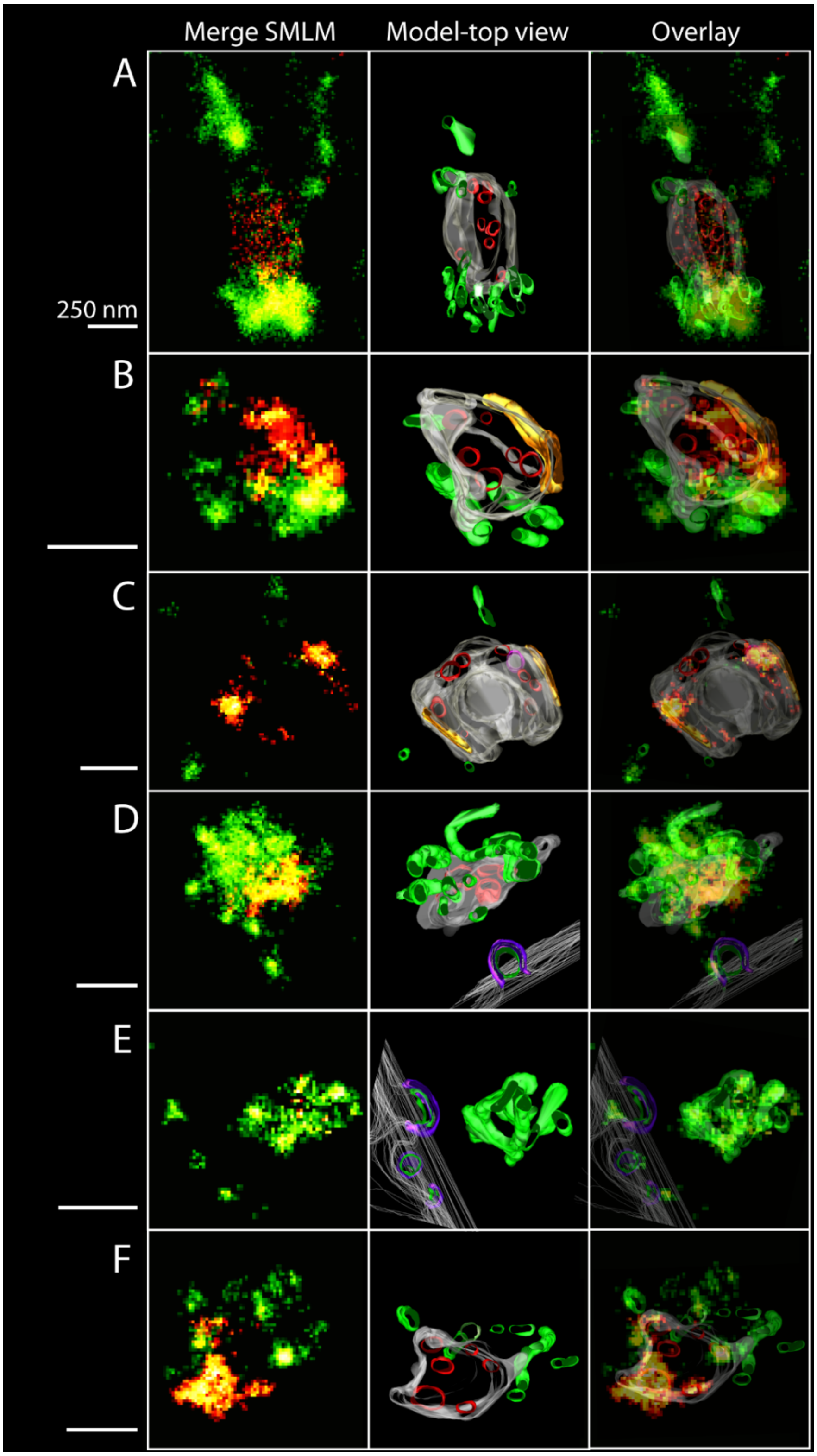

Figure S12: Direct overlays of the merged SMLM images and the corresponding coloured ultrastructural models presented in Figure 6.

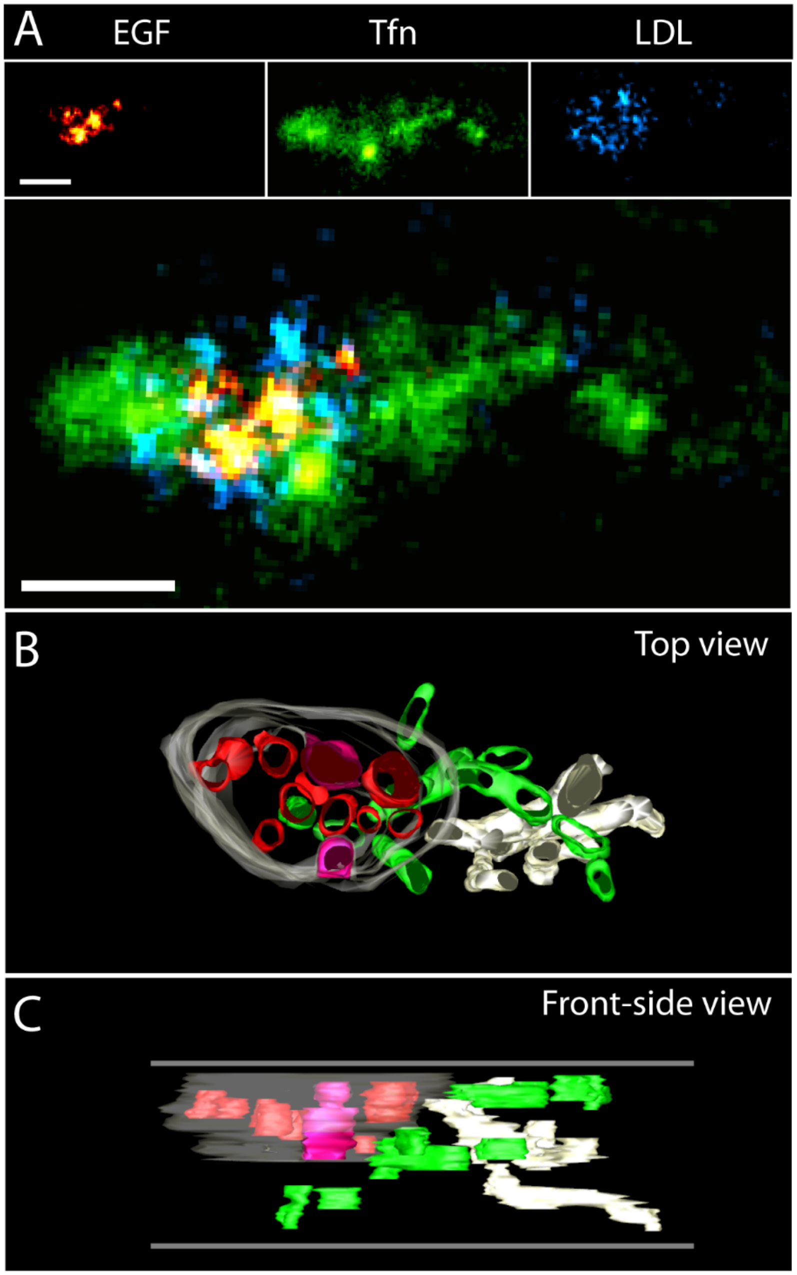

Figure S13: Compartmentalization of EGF, Tfn and LDL on endosomes visualized by triple-colour CLEM using semi-thin Tokuyasu sections.

(A) SMLM data for EGF-Alexa647 (red), TFn-Alexa568 (green) and LDL-Alexa488 (blue). (B,C) Ultrastructural models of the endosome based on a tomogram reconstructed from double-axis tilt series. Colours used in models represent: (grey) limiting membrane, (red) ILV, (magenta) ILV continuous with the limiting membrane, (green) recycling tubules. LDL is localized to the lumen of the central vesicle. Horizontal grey lines in (C) indicate top and bottom sides of a reconstructed tomogram. Scalebar: 250 nm.

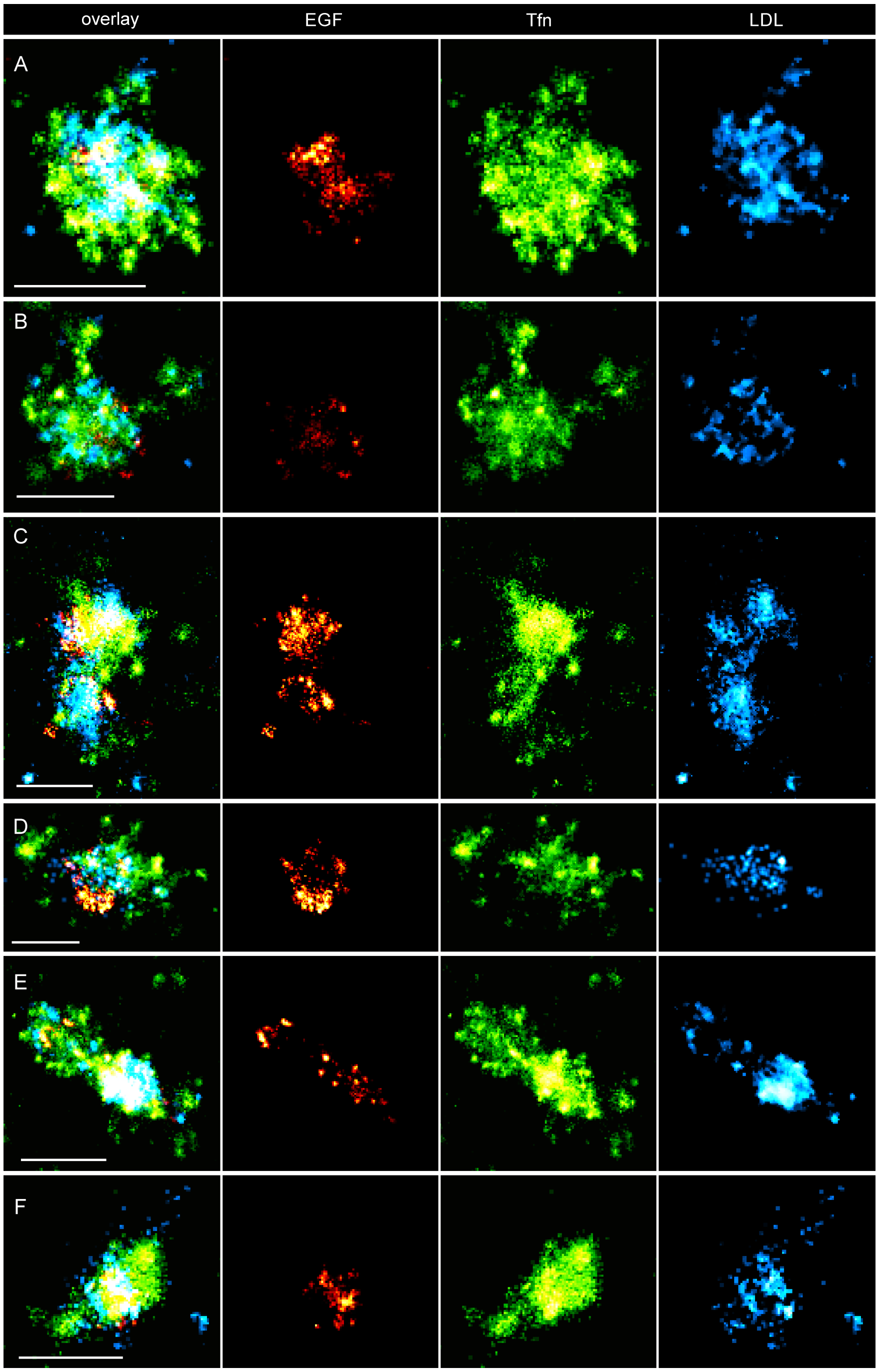

Figure S14: Compartmentalization of EGF, Tfn and LDL on endosomes visualized by triple-colour SMLM. EGF-Alexa647 (red), TFn-Alexa568 (green) and LDL-Alexa488 (cyan). Scalebars: 500 nm.

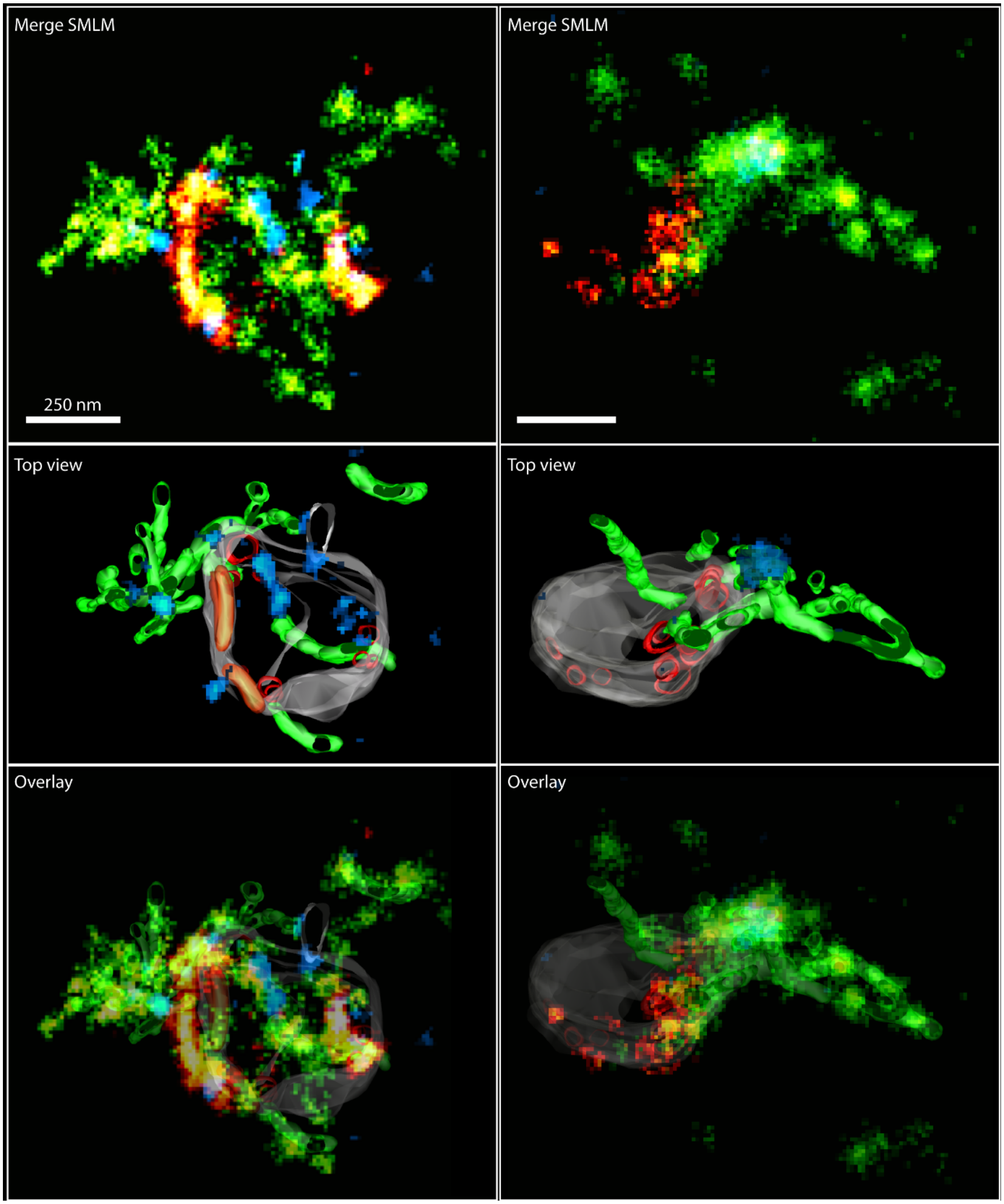

Figure S15: Direct overlays of the merged SMLM images and the corresponding coloured ultrastructural models presented in Figure 7.

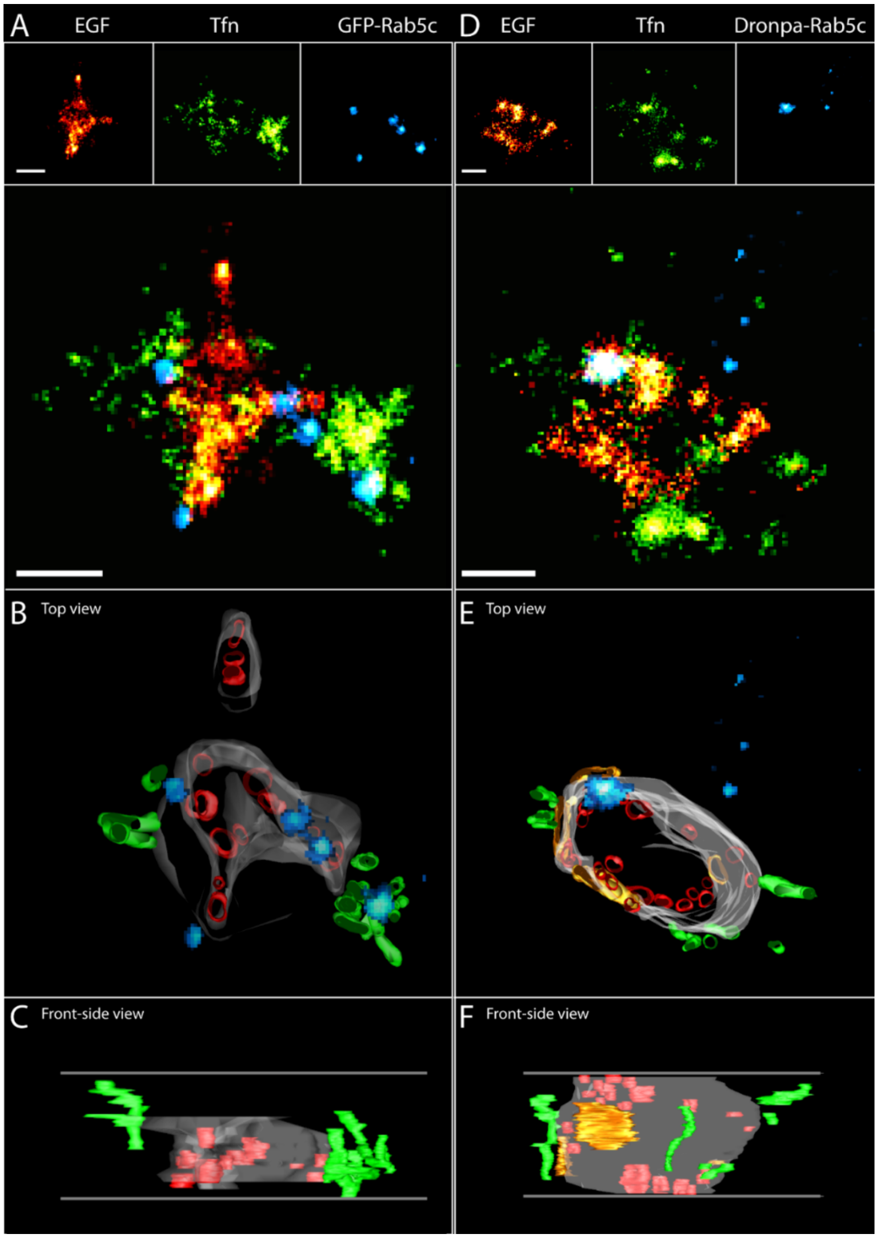

Figure S16: Mapping of Rab5c on endosomes visualized by triple-colour superCLEM (additional examples).

Mapping of Rab5c on endosomes visualized by triple-colour superCLEM using semi-thin Tokuyasu sections. (A,D) SMLM data for EGF-Alexa647 (red), TFn-Alexa568 (green) and GFP/Dronpa-Rab5c (blue). (B,C,E,F) Ultrastructural models of endosomes based on tomograms reconstructed from double-axis tilt series. Colours used in models represent: (grey) limiting membrane, (red) ILV, (green) recycling tubules, (orange) sorting microdomains (SμD), (blue) Rab5c. Horizontal grey lines in the bottom images (C,F) indicate top and bottom sides of reconstructed tomograms. Scalebar: 250 nm.

localization precision

	647 nm excitation	561 nm excitation	488 nm excitation
(A) whole cell	7.95 nm	13.25 nm	14.78 nm
(B) sections on glass	9.06 nm	11.58 nm	12.89 nm
(C) sections on EM grid	12.60 nm	13.91 nm	16.27 nm

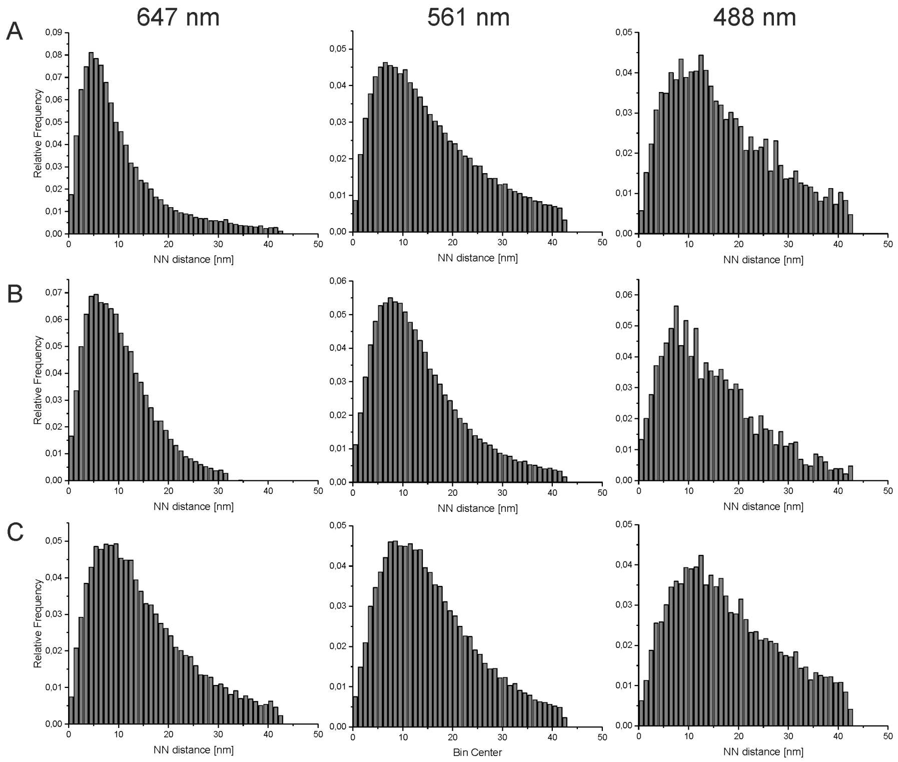

Figure S17: Localization precision for individual SMLM colour channels in different sample situations. Above: tabular overview of the calculated localization precisions in different sample settings (rows) and excitation channel (columns). Below: Distributions of nearest-neighbour (NN) distances of localizations in consecutive frames in different sample settings (rows, (A) whole cell, (B) sections on glass, (C) sections on EM grids (superCLEM)) and excitation channel (columns).

1. Royer LA, Weigert M, Günther U, et al. ClearVolume: open-source live 3D visualization for light-sheet microscopy. *Nature Methods*. 2015;12(6):480-481. doi:10.1038/nmeth.3372
